# Reflecting on Advances in Lower Extremity Biomechanics and Injury Prevention: Insights from Our Special Issue

**DOI:** 10.3390/sports12010013

**Published:** 2023-12-29

**Authors:** Jesper Augustsson

**Affiliations:** Department of Sport Science, Faculty of Social Sciences, Linnaeus University, 39182 Kalmar, Sweden; jesper.augustsson@lnu.se; Tel.: +46-(0)705589752

## 1. Introduction

It is with great pleasure that we present this Editorial, marking the completion of our Special Issue on Lower Extremity Biomechanics and Injury Prevention. Over the course of this collection, we have had the privilege of exploring a diverse array of research contributions that will significantly enhance our understanding of the intricate relationship between biomechanics and injury risk in athletes [1].

## 2. Advancements in Technology

In this Special Issue, motion capture systems played a pivotal role in unraveling the complexities of dynamic movements such as running, jumping, and cutting [2]. These technological advancements have empowered researchers to delve into joint angles, forces, and movements, providing unprecedented insights into movement patterns associated with increased injury risk [3].

## 3. Strength Training Insights

Contributions in strength training research have been particularly illuminating. The examination of exercises like the barbell squat and Nordic hamstring exercise has not only elucidated their impact on lower extremity strength and power but has also underscored their potential in reducing injury risk when executed correctly [4]. The practical applications of strength training in injury prevention add a tangible dimension to our collective knowledge, bridging the gap between theory and athletic performance.

## 4. Identifying Gaps and Future Directions

While celebrating the strides made, it is crucial to acknowledge the gaps that persist in our understanding of effective lower extremity injury prevention [5]. This Special Issue serves as a call to action, urging researchers to continue pushing boundaries and exploring uncharted territories. By inviting contributions ranging from practical applications to advanced biomechanical analyses, we strive to foster a comprehensive understanding that will ultimately inform evidence-based strategies for preventing lower extremity injuries in athletes [6].

## 5. Acknowledgments and Gratitude

The success of this Special Issue would not have been possible without the dedication and expertise of the contributing authors, reviewers, and our esteemed Editorial Board. Each paper represents a valuable piece in the puzzle, contributing to the broader narrative of advancing sports science and athlete well-being.

In conclusion, this Special Issue stands as a testament to the collaborative efforts of the research community in advancing our knowledge of lower extremity biomechanics and injury prevention. As we move forward, let this collection inspire further inquiry and innovation, propelling us toward a future where athletes can perform at their best, supported by evidence-based practices.
